# Trait Aggressiveness Is Not Related to Structural Connectivity between Orbitofrontal Cortex and Amygdala

**DOI:** 10.1371/journal.pone.0101105

**Published:** 2014-06-30

**Authors:** Frederike Beyer, Thomas F. Münte, Juliana Wiechert, Marcus Heldmann, Ulrike M. Krämer

**Affiliations:** Dept. of Neurology, University of Lübeck, Lübeck, Germany; University of Medicine & Dentistry of NJ - New Jersey Medical School, United States of America

## Abstract

Studies in both pathological and healthy samples have suggested altered functional connectivity between orbitofrontal cortex (OFC) and amygdala as a possible cause of anger and aggression. In patient populations presenting with pathological aggression, there is also evidence for changes in structural connectivity between OFC and amygdala. In healthy samples, however, the relationship between white matter integrity and aggression has not been studied to date. Here, we investigated the relationship between trait aggressiveness and structural OFC-amygdala connectivity in a large sample (n = 93) of healthy young men. Using diffusion tensor imaging, we measured the distribution of fractional anisotropy and mean diffusivity along the uncinate fascicle bilaterally. We found no differences in either measure between participants high and low in physical aggressiveness, or between those high and low in trait anger. Our results therefore argue against a direct relationship between structural OFC-amygdala connectivity and normal-range trait aggressiveness.

## Introduction

Inhibitory control of amygdala activity by prefrontal structures is a crucial aspect of emotion regulation and dysfunction in this network is associated with anxiety-related disorders [1] as well as aggression [2]. Altered functional connectivity between the prefrontal cortex, especially the medial orbitofrontal cortex (mOFC), and the amygdala has been shown to be related to aggressive tendencies in pathologically aggressive patients and healthy samples [3], [4]. Accordingly, integrity and strength of structural connections between medial prefrontal cortex and amygdala might equally be related to interindividual variability in aggression.

Reciprocal connections between prefrontal cortex and amygdala exist anatomically via the uncinate fascicle (UF), which connects prefrontal cortex and the anterior temporal lobe [5], [6]. Research in rhesus monkeys suggests that anatomical connections between prefrontal cortex and amygdala are highly reciprocal, i.e. areas of prefrontal cortex receiving strong input from the amygdala also possess a high density of efferent fibers projecting back to the respective amygdala region [7]. While evidence exists that the UF is associated with the transmission of socio-emotional information [6], research in this domain and especially concerning specific functions such as control of aggressive impulses is still scarce and inconclusive.

Diffusion weighted tensor imaging (DTI) is a non-invasive imaging method allowing for in-vivo measurement of fiber tracts in the brain. Measuring the diffusion of water within each voxel, different parameters can be estimated with DTI. Fractional anisotropy (FA) reflects the coherence of diffusion in a given voxel. Mean diffusivity (MD), on the other hand, is inversely related to the quality of white matter connections [8].

Several studies using DTI have shown a relationship between alterations in prefrontal-subcortical anatomical connections or prefrontal white matter volume and aggression-related psychopathology. Psychopathic men show reduced FA-values in the right UF, and the number of streamlines in the bilateral UF is negatively related to antisocial behavior in psychopaths [9], [10]. In adults with antisocial personality disorder, FA-values were found to be decreased in a range of brain areas including the right UF. MD-values were increased in the right UF, among other regions [11]. Schizophrenic men presented with a negative relationship between inferior frontal FA-values and motor impulsivity, and with a positive relationship between inferior frontal diffusivity and aggression scores [12].

In adolescents (mean age  = 18.4 years) with conduct disorder (CD), who present with aggressive and antisocial behavior, increased FA-values were found in the UF [13]. As white matter increases into early adulthood [14], the authors interpret this finding as a reflection of premature maturation of this fiber tract in adolescents with CD. However, another study investigating youths with conduct disorder and psychopathic traits found no difference in FA-values in the UF compared to healthy controls [15]. The authors argue that since the investigated sample was rather young (mean age 14.3 years), impairments in structural amygdala-orbitofrontal connectivity in CD patients may develop during the maturation of white matter across adolescence and early adulthood.

Evidently, the reviewed literature is based on patient populations presenting with complex syndromes, precluding any definite conclusions concerning the importance of the UF for the control of anger or aggression. If information transmitted by this fiber tract is important for social information processing involved in the development of non-aggressive behavioral strategies and emotional regulation, this might also be reflected in healthy samples.

In this study, we investigated the relationship between measures of diffusivity in the UF and trait aggressiveness in healthy young men. If strength of the UF indeed is of specific importance for control of anger and aggression, a negative relationship between anger and aggression scores and FA-values would be expected, with a reverse relationship between MD-values and aggression scores.

## Methods

### Participants

99 healthy male volunteers were recruited for this study. All participants were free of psychiatric and neurological disorders (self-report). Since differences between men and women have been shown both for white matter properties [16], [17], [18] and physical aggression scores [19], we limited our study to male participants. Of the investigated subjects, 5 were excluded from data analysis due to artifacts in their DTI-images or anatomical abnormalities. Data from one participant was excluded because the AFQ tractography failed due to a low number of original tracts identified in the whole brain tractography step (see below for detailed information on the tractography). Thus, data of 93 participants was included in the analysis. Mean age of the participants was 23.2 years (SD  = 2.7) and all but 7 were right handed.

A-priori estimations of required sample size were difficult, as there is little evidence for the effect sizes to be expected in healthy samples. With n = 93, α = .005 (to account for multiple comparisons) and β = .8, the minimum effect size detectable is ρ = .72. This is considerably smaller than the effects in the above-cited studies comparing pathological and normal populations, which showed effect sizes between 1.25 and 2.5 [9], [11], [13].

The study was approved by the Ethics Committee of the University of Lübeck and performed according to the Declaration of Helsinki. Written informed consent was obtained from all participants.

### Trait Aggression measure

As a measure of trait aggression, participants filled out a German version of the Buss and Perry Aggression Questionnaire (AQ) [20], which comprises four subscales: anger, physical aggression, verbal aggression and hostility. Since we hypothesized that deficits in prefrontal-amygdala connectivity should be specifically associated with emotional dysregulation and aggressive behavior, we focused our analysis on the anger and physical aggression subscales. The physical aggression scale contains items such as “If somebody hits me, I hit back” or “I have become so mad that I have broken things.” The anger scale contains items such as “I flare up quickly but I get over it quickly” or “Some of my friends think I'm a hothead.” Answers in the AQ are given on a 5-point Likert scale, with high scores representing high aggressiveness [19].

### Behavioral Aggression measure

As an additional aggression measure, we used behavioral data derived from the Taylor Aggression Paradigm (TAP) [21] in two subsamples of our subjects. The TAP is set up as a competitive reaction time task in which the participant competes against an ostensible opponent in multiple rounds. In each round of the TAP, the participant must press a button as quickly as possible in response to a visual cue and try to be faster than his opponent. The loser of each round is punished with an aversive stimulus, the intensity of which is determined by the winner. Both outcome of the reaction time task and the opponent's punishment selection are under control of the experimenter. Thus, in the TAP, the punishment selected by the opponent serves as a method for provocation and the punishment selected by the participant serves as a measure of aggression.

We implemented the TAP such that at the beginning of each round, the participant selected a punishment level for his opponent. This was followed by the reaction time task and an outcome phase, during which the participant was informed whether he had won or lost and which punishment level his opponent had selected. As a punishment stimulus, we used an aversive noise that could be adjusted in terms of loudness on a scale from 1–8. In lost trials, the noise was played in the respective loudness at the end of the outcome phase. The outcome of the reaction time task, and also the opponent's selections, were controlled by the computer. The mean punishment level selected by the participant was used as a measure of aggressive behavior.

For a subsample of 34 participants, we used behavioral data from a version of the original TAP by Taylor: The opponent started with low punishment levels (1–2) and ended with the highest selection, being 8 (mean  = 4.0), shuffled in 4 blocks (means  = 1.5/3.5/5.5/7.5, ranges  = 1–2, 3–4, 5–6, 7–8). Each participant played 24 trials against the confederate and won 50% of them. As behavioral aggression measure for each participant, we used the mean punishment selection across all trials.

For a second subsample of 33 participants, we used behavioral data from a version of the TAP adapted for functional MRI and including video sequences showing the ostensible opponent. The video sequences were presented at the beginning of each decision phase and showed the opponent bearing a neutral or angry facial expression. The TAP consisted of 60 trials which were divided into three blocks, and the opponent selected medium to high punishments (3–8, mean  = 4.8). The fMRI results of this study will be presented elsewhere [22]. For the present purpose, we only considered the mean punishment selection across all trials as behavioral measure of aggression.

For both subsamples, the behavioral aggression measure used reflects the participants' propensity to aggress in situations that include some form of provocation. All participants were confronted with an opponent who at least in some trials selected high punishments. However, there were also differences both in the mean punishment level participants received and the general setup of the task (in subsample 2, the task was adapted for fMRI and thus longer, and included video sequences). Thus, while the two samples could not be investigated as one group, both behavioral measures reflect reactive aggressive behavior in a competitive interactive context.

### DTI data acquisition

Images were recorded on a Philips Achieva 3-T scanner (Philips Healthcare, the Netherlands) with a standard 8-channel head coil. Diffusion weighted images (1 image without diffusion gradients and 32 images with diffusion gradients applied) were recorded with the following specifications: 70 axial slices; slice thickness 2 mm; in-plane voxel size 2×2 mm; single-shot EPI sequence; TR  = 7852 ms; TE  = 60 ms; flip angle 90°; FOV  = 224 mm; matrix  = 112×112. High resolution structural images were obtained applying a T1-weighted 3D turbo gradient echo sequence with SENSE (FOV  = 240 mm; matrix  = 240×240; 180 sagittal slices of 1 mm thickness).

### Data analysis

We used the open-source software FSL 5.0 (http://neuro.debian.net/pkgs/fsl-5.0.html#binary-pkg-fsl-5-0) [23] for eddy correction and extraction of diffusion tensors from the original diffusion weighted images. We also used FSL's brain extraction tool to skull-strip DTI (probability-cutoff 0.3) and anatomical images (cutoff 0.5).

For further analysis, we used the Automated Fiber Quantification (AFQ) toolbox (http://white.stanford.edu/newlm/index.php/AFQ). This is an open-source matlab-based DTI analysis tool built to automatically identify the 20 major fiber tracts in individual DTI-images in their native space. Details on the methods implemented by the AFQ-toolbox can be found in [24]. Briefly, analysis with AFQ consists of the following processing steps: first, the eddy-corrected, skull-stripped DTI-images were imported into AFQ format and aligned with the respective skull-stripped T1-weighted image. Then, deterministic fiber-tracking was performed across the whole brain and subsequently, for each of the 20 target tracts tract segmentation was performed based on two waypoint-regions of interest (ROIs). These tracts were then refined and cleaned by removing aberrant fibers. Further analysis focused on the portion of the tract between the two waypoint ROIs. This section was divided into 100 equally spaced nodes, such that FA- and MD-values, or other values of interest, could be investigated for each node separately rather than averaged across the whole tract.

Using this method, Yeatman et al. [24] were able to show systematic variations in FA-values along fiber tracts that were consistent between subjects. They found that developmental changes of FA-values vary along fiber tracts, underlining the importance of investigating separate nodes rather than mean-FA-values for entire tracts. Furthermore, they demonstrated the validity of this method by replicating previous findings of correlations between reading skills and FA-values in children.

For each subject, we extracted the FA- and MD-values for the left and right UF. The UF is defined by a seed ROI in the anterior temporal lobe and an end-ROI close to the OFC. We then used SPSS to analyze the relationship between DTI-values and AQ scores.

Since the scores on the anger and physical aggression scales were not normally distributed (Kolmogorov-Smirnov-test p<.001), we did not perform correlational analyses on the data. Instead, we performed a median split based on the physical aggression subscale and compared FA- and MD-values between high and low aggressive participants using independent sample t-tests for each of the 100 nodes of the UF in each hemisphere. We then followed the same procedure for the anger subscale.

As an exploratory approach, we also compared FA-values between high and low aggressive participants for the remaining 18 fiber tracts. Note that as not all tracts can be detected reliably in all participants, these comparisons were based on slightly different group sizes (range: 86–93).

For the behavioral aggression data, we correlated mean aggression scores from the TAP with FA-values for the left and right UF. This was done separately for the two groups for which we had data from different versions of the TAP.

We accepted group differences as significant if they survived a p-value cutoff of 0.05, false-discovery-rate (FDR-) corrected. For the UF, we performed FDR-correction separately for the left and right tract. For the exploratory whole-brain analysis, we corrected for all calculated tests (18 tracts with 100 nodes each).

## Results

**Figure 1:**
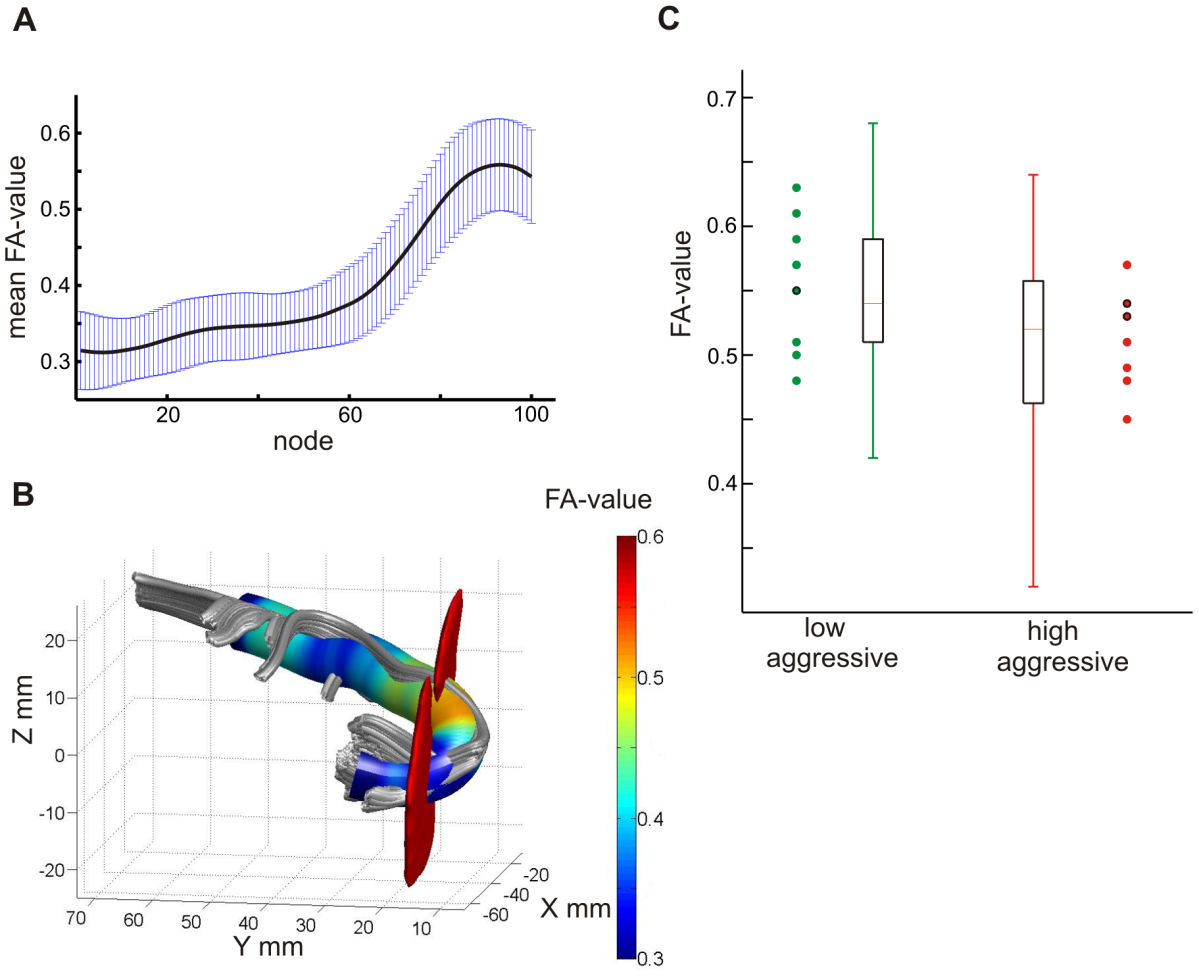
Descriptive results. The distribution of mean FA-values along the left uncinate fascicle (UF) between the tract defining regions of interest (ROIs) is shown (**A**). Standard deviations for each of the 100 nodes are shown in blue. **B** shows the distribution of FA-values along the left UF for a representative subject. Tract-defining ROIs are depicted in red. **C** shows mean FA-values for the low and high aggressive groups as defined by the median split based on physical aggression scores for node 83 of the left uncinate fascicle. Single subject FA-values are depicted for the nine participants with the lowest and highest aggression scores. Data points encircled in black represent two cases.

Along the left UF, mean FA-values ranged from 0.31–0.56 (SD 0.039–0.066). As can be seen in figure 1A, mean FA-values increased along the tract from the ROI in the temporal lobe towards the prefrontal ROI. Figure 1B shows the distribution of FA-values along the left UF in a representative subject. These results were similar for the right UF (means 0.32–0.55; SD 0.032–0.050) and are consistent with previous findings concerning the distribution of FA-values along the UF [24].

Mean MD-values along the left UF ranged from 0.75–0.89 (SD 0.033–0.072). Along the right UF, mean MD-values ranged from 0.74–0.83 (SD 0.032–0.043).

The median of the physical aggression scores was 1.67, with quartiles 1.38 and 2.11 (mean score  = 1.85). The median of the anger scores was 2.0, with quartiles 1.57 and 2.5 (mean score  = 2.16).

### UF properties and physical aggression

Participants with a physical aggression score of 1.63 and lower were classified as low aggressive, resulting in a 46∶47 ratio of low: high aggressive participants (mean aggression scores 1.36 and 2.32; SD  = 0.7).

Low and high aggressive participants did not differ significantly in their FA- or MD-values for the UF in either hemisphere. With a more lenient significance threshold of p<.05 uncorrected, we observed higher FA-values for the low aggressive group bilaterally in the anterior part of the UF, close to the prefrontal ROI. In the left UF, group differences were observed for nodes 76–95, in the right UF for nodes 89–95, as well as nodes 97, 99 and 100. This trend for statistical differences might point to “true” personality effects which are diminished by the rather rough approach of a median-split. To ensure that we did not miss a true effect, we further investigated the distribution of FA-values on a descriptive level. A true personality effect on FA values should be clearly observable in participants with extreme trait scores. For the node with the greatest difference between groups (node 83 in the left UF; means  = 0.547 and 0.511), we therefore plotted the distribution of FA-values for the low and high aggressive groups together with the FA-values of the nine participants with the highest and lowest aggression scores. As can be seen in figure 1C, there was no consistent trend of highest aggression being associated with lowest FA-values or vice versa, supporting the assumption that the observed group differences in FA-values constitute a chance finding.

### UF properties and anger

The critical value for the median split was 2.0. Since 41 participants had an anger score below and 42 participants had an anger score greater than this, we excluded participants with an exact score of 2.0 (n = 10) in order to yield comparable group sizes (mean scores  = 1.56 and 2.75; SD  = 0.71).The groups high and low in trait anger did not differ in their FA- or MD-values across the UF in either hemisphere.

For the remaining 18 fiber tracts, we also did not observe any significant effects for trait physical aggression or trait anger.

### UF properties and aggressive behavior

For the behavioral variant of the TAP, mean punishment selection was 3.80 (SD  = 1.33; n = 34). For the fMRI-variant of the TAP, we observed a mean punishment selection of 4.3 (SD  = 0.86; n = 33).

In support of our results for the AQ, we found no significant correlations between aggressive behavior and FA-values in the left or right UF.

## Discussion

This study revealed that in healthy participants, integrity of the bilateral UF is not related to trait anger or physical aggressiveness. Thus, it appears that deficits in structural prefrontal-amygdala connectivity in aggressive individuals [9], [10] are specific to pathological populations such as criminal offenders with psychopathy or adolescents and adults with conduct disorder.

The large sample size of our study makes it unlikely that we missed a true effect due to lack of power. It is important to note, however, that we only investigated men in the age range of 18–30 years, with a mean age of 23. Studies investigating changes in the UF in pathologically aggressive groups found no difference in FA-values in children around 14 years of age [15], increased FA-values in adolescents around 18 years [13] and decreased FA-values in adults [11]. As white matter maturation continues into early adulthood [14] it is possible that differences in the speed of white matter maturation within our sample eliminated effects of trait aggressiveness. Nevertheless, our results argue against a direct relationship between trait aggressiveness and integrity of the UF in healthy male participants. Such a relationship should be reflected in lower UF maturation in high aggressive individuals, which we did not observe.

Other studies in healthy participants have found significant correlations between FA-values and trait anxiety [25], behavioral approach motivation [26] and motor learning [27]. Thus, we do not believe that our results are based on a general lack of variability in DTI-measures in healthy samples or insensitivity of such measures to personality traits.

Regarding aggressive behavior, however, Finger et al. [15] in their study on younger adolescents concluded that functional connectivity between OFC and amygdala is a more sensitive measure of maladaptive development than structural connectivity. Decreased functional connectivity between amygdala and prefrontal cortex in response to negative social stimuli has been observed in adolescents with CD [28], adults with impulsive aggression [4], and also healthy participants high in approach motivation [3]. Accordingly, investigating functional connectivity in healthy samples might reveal more subtle differences between low and high aggressive individuals.

### Limitations

It has to be stressed that our participants were mostly college students and as such a rather homogenous sample. Physical aggression and anger scores were not normally distributed with a bias towards low scores, precluding the conductance of correlation analyses. We cannot assume, therefore, that our participants reflected the entire range of trait aggressiveness that one might observe in the general population. In a more representative sample, we would expect more extreme values in aggression scores, which then might be related to properties of the UF. In his validation study for the German version of the AQ, Herzberg [20] found higher mean values for the physical aggression and anger scales than we did (2.41 and 2.64 respectively, vs. 1.85 and 2.16 in our study). Notably, the sample in this validation study was not limited to university students. Thus, our results argue against a relationship between UF integrity and aggressiveness in presumably high-functioning and self-controlled young men, but they cannot be generalized to all men of this age group. Future studies should investigate samples recruited from a broader population in order to allow for correlational analyses to account for the whole spectrum of variance in white matter properties and aggression scores. Alternatively, specifically targeting extreme groups involved in socially accepted aggressive behavior, such as high-contact sports might increase effect sizes in group comparisons.

Another possible limitation of this study is that across the entire sample, we only used a self-report questionnaire as measure of aggression. However, further investigating the relationship between FA-values and aggressive behavior in the TAP for sub-samples, we could confirm the null-findings. Thus it seems unlikely that our results are due to a bias in self-report.

## Conclusions

While pathological aggression has been associated with decreased structural connectivity between amygdala and OFC, this does not seem to be the case in healthy men. According to our results, between-subject variability in trait physical aggression and anger in young men is unrelated to fractional anisotropy along the uncinate fascicle connecting anterior temporal and prefrontal areas.

## Acknowledgments

We thank Susanne Schellbach for help during data acquisition.
